# Impact of parent-child separation on children’s social-emotional development: a cross-sectional study of left-behind children in poor rural areas of China

**DOI:** 10.1186/s12889-021-10831-8

**Published:** 2021-04-29

**Authors:** Huifeng Shi, Yuanyuan Wang, Mengshi Li, Chang Tan, Chunxia Zhao, Xiaona Huang, Yan Dou, Xiaoqian Duan, Yufeng Du, Tianchen Wu, Xiaoli Wang, Jingxu Zhang

**Affiliations:** 1 Department of Maternal and Child Health, Peking University School of Public Health, 38 Xueyuan Road, Haidian District, Beijing, 100191 China; 2 Department of Obstetrics and Gynecology, Peking University Third Hospital, 49 North Garden Road, Haidian District, Beijing, 100191 China; 3 National Clinical Research Center for Obstetrics and Gynecology, Peking University Third Hospital, 49 North Garden Road, Haidian District, Beijing, 100191 China; 4 Section of Health, Nutrition, and Water, Environment and Sanitation, UNICEF China, 12 Sanlitun Road, Chao Yang District, Beijing, 100600 China; 5 Institute of Epidemiology and Statistics School of Public Health, Lanzhou University, 199 West Donggang Road, Lanzhou, 730000 China; 6 National Health Commission Key Laboratory of Reproductive Health, 38 Xueyuan Road, Haidian District, Beijing, 100191 China

**Keywords:** Parent-child separation, Left-behind children, Social-emotional development, Early childhood

## Abstract

**Background:**

Parent-child separation is a considerable adversity for left-behind children (LBC), but there is little evidence on the association between detailed characteristics of parent-child separation and social-emotional development among LBC. This study examined the characteristics of parent-child separation and its impacts on developmental delay among under-3 LBC in poor rural China.

**Methods:**

We used data from 811 LBC surveyed in five poor counties in rural China in 2018. Detailed characteristics of their parental migration were recalled by their primary caregivers in face-to-face interviews. The children’s social-emotional development was measured by using the Ages and Stages Questionnaire: Social-Emotional. Logistic regression was employed to examine the association of detailed characteristics of parent-child separation with early social-emotional problems after adjusting for the children’s and primary caregivers’ sociodemographic characteristics.

**Results:**

287 (35.4%) children were left behind by fathers and cared for by mothers (FM-MC), while 524 (64.6%) were left behind by both parents and cared for by grandparents (PM-GC). The rate of social-emotional problems among LBC was 36.8% (PM-GC vs FM-MC: 40.6% vs 29.5%; aOR 1.51, 95% CI: 1.06 to 2.16). For paternal migration, the medians of the child’s age at the first migration and average duration per migration were 3 months (IQR: 1 to 9 months) and 4.48 months (IQR: 2.38 to 7.54 months), respectively. For maternal migration, the corresponding values were 9 months (IQR: 6 to 13 months) and 4.65 months (IQR: 2.71 to 7.62 months), respectively. On average, LBC had been separated from fathers for 72% of their life due to paternal migration and from mothers for 52% of their life due to maternal migration. No significant association was found between the detailed characteristics of paternal migration and social-emotional development among LBC, while social-emotional problems among LBC were significantly associated with the proportion of cumulative duration of maternal migration in the child’s lifetime (aOR 2.83; 95% CI: 1.13 to 7.10).

**Conclusions:**

LBC under 3 years had a high risk of social-emotional problems in poor rural China. Cumulative exposure to maternal migration may be detrimental to LBC’s early social-emotional development. Programs are necessary to support these children as well as their families.

**Supplementary Information:**

The online version contains supplementary material available at 10.1186/s12889-021-10831-8.

## Background

Internal migration is an increasing issue globally. Most labour migrants migrate from rural to urban settings, resulting in a growing number of left-behind children when their parents migrate, particularly in low-income or middle-income countries (LMICs). In China, approximately 40 million **(**38%) rural children are left behind by one or both migrant parents, accounting for approximately 15% of all children in the entire country [1]. The proportion of left-behind children in rural settings is 40% in South Africa, 36% in Ecuador, and 27% in the Philippines [2]. The previous literature has revealed high risks of poor nutrition, well-being, and mental health among left-behind children [2–4].

Children’s social-emotional development provides them the capacity to experience, manage and express both positive and negative emotions, develop a satisfying relationship with other children and adults, and actively explore their environment and learn [5]. Early childhood, especially the first 3 years of life, is characterized by rapid and dynamic brain development, which is a critical developmental stage for an individual’s social and emotional functions or disorders throughout their life span [6, 7]. Social-emotional development in early childhood is markedly shaped by different aspects of the parent-child relationship and different underlying mechanisms [8]. Among left-behind children, their development may be adversely affected by interrupted attachment and weakened nurturing care due to parent-child separation [9]. Early age-appropriate stimulation and responsive care shape the brain architecture by forming, strengthening, and pruning synaptic connections [10], while recurrent or chronic violent discipline, neglect, and abuse can continuously stimulate the stress response system and then adversely affect brain development [11, 12]. The effects of these early adverse experiences and trauma may persist into adulthood [13, 14]. Previous evidence has demonstrated that children who were separated from their parents at a younger age, particularly under the age of 3 years, had higher risks of physical, mental and developmental disorders in the childhood and even adulthood periods [15–19]. Theoretically, the timing of parent-child separation can be mainly characterized in three domains: the child’s age at the first separation, the average separating duration per time, and the cumulative separating duration. However, despite a growing body of research in this field, there are limited data to depict such characteristics of parent-child separation and to determine their association with social-emotional development among left-behind children in early childhood.

Based on the baseline survey of the Rural Left-Behind Children Health and Development Program (RLBCHD), launched by the United Nations Children’s Fund (UNICEF), this study aimed to explore the characteristics of parent-child separation and its impacts on developmental delay among under-3 children who were left behind in poor rural China and then to develop comprehensive intervention strategies and evidence-based policies to improve rural left-behind children’s early development and well-being.

## Methods

### Design and participants

We used cross-sectional data from the baseline survey of UNICEF’s RLBCHD Program prior to the intervention [9], which was conducted between April 2018 and July 2018 in five poor counties from five different provinces in eastern and western China: Yudu County, Jiangxi Province; Sansui County, Guizhou Province; Tongjiang County, Sichuan Province; Lushi County, Henan Province; and Pingshan County, Hebei Province. In four counties (Yudu, Sansui, Tongjiang and Lushi), a multistage stratified sampling method (according to the number of children under 3 years) was used to select program villages. In Pingshan County, all program villages were included due to the limited number of left-behind children under 3 years of age. To evaluate the effects of the RLBCHD program, a control village from the same county was matched to each of the sampled program villages according to the number of children under 3 years, per capita income, and distance to the county capital. Finally, 113 sampling villages from 27 towns were included in the baseline survey. The primary caregivers of left-behind children in these villages were invited to participate in a face-to-face questionnaire survey. Written informed consent was obtained from the caregivers prior to participation in the survey.

This study focused on left-behind children with migrated fathers only and being cared for by their mothers (FM-MC), and those with both mother and father migrated and being cared for by their grandparents (PM-GC). We did not include left-behind children with migrated mothers only (*n* = 12) or other types of parental migration-caregiving arrangements (*n* = 34) due to the small sample size. Finally, 811 left-behind children were eligible for inclusion in the analysis. Among them, 759 were measured by using the Ages and Stages Questionnaire: Social-Emotional (ASQ:SE) to evaluate their social-emotional development. We did not measure 17 children under three 3 months because they were less than the measurement age of the ASQ:SE and we failed to measure 35 children aged 3–35 months because their caregivers were eager to do housework or the interviews could not continue due to the children crying.

### Procedures and measures

Face-to-face interviews with primary caregivers of left-behind children were conducted by uniformly trained local health workers. Data were collected, input, and saved by means of an electronic questionnaire application, with logic and integrity-checking functions that were preset by the investigators.

1. *Child and primary caregiver characteristics and family economics*. Child sex, birthday, and birth weight were collected according to the child’s birth certificate or immunization record. Data about the primary caregivers’ relationships with the children, sex, age, ethnicity, education, and whether they had assistant caregiver(s) were collected. Depressive symptoms among the primary caregivers were measured by using Zung’s Self-rating Depression Scale (ZSDS), which consists of 20 items with a total score ranging from 20 to 80 (a ZSDS score of ≥50 was defined as depression) [20, 21]. Family economics was measured by the types of household electrical appliances and vehicles owned, including a television, rice cooker, washing machine, refrigerator, air conditioner, computer, motorcycle/tricycle, and automobile (*n* < 3 was defined as ‘poor household wealth’) (See Supplementary file) [22].

(2) *Characteristics of parental migration*. Investigators asked the primary caregivers to recall (a) the child’s age of months at the first paternal and maternal migration, (b) migration and return times, and (c) average duration per parent-child reunion (See Supplementary file). Cumulative migrating duration was calculated by subtracting (a) + (b) * (c) from the child’s age of months at the survey. The average duration per migration was calculated by dividing the cumulative migration duration by the migration time. The proportion of cumulative migrating durations in the child’s lifetime was calculated by dividing the cumulative migrating duration by the child’s age in months at the time of the survey.

(3) *Child’s social-emotional development*. The children’s social-emotional development was measured by using the Ages and Stages Questionnaire: Social-Emotional (ASQ:SE). The ASQ:SE includes eight developmental screening questionnaires at different ages and stages for children between 3 and 66 months of age. Each questionnaire consists of 19–33 items, and each item is rated on a three-point Likert scale (10 = most of the time, 5 = sometimes, 0 = never or rarely; negative wording was scored reversely). Children with a total score equal to or exceeding the cut-off point were defined as ‘at risk’, indicating that the child needs further evaluation and/or intervention. The cut-off point for each age group has been adapted for the Chinese population [23, 24].

### Statistical analysis

We used proportions to describe the children and primary caregivers’ characteristics measured by categorical variables and the χ^2^ test to compare the differences between children with both migrated fathers and mothers and those with migrated fathers only. We calculated medians and interquartile ranges (IQRs) for the children and primary caregivers’ age, child age at the first parental migration, average duration per parental migration, and proportion of cumulative durations of parental migration in the child’s lifetime, and we used the Mann-Whitney U test to compare the differences among children with various types of parental migrations. Furthermore, we used histograms to present the probability density distribution of the child’s age at the first paternal and maternal migration. We fitted Loess regressions to explore the association of average duration per parental migration and the proportion of cumulative durations to parental migration with child age in months.

Logistic regression was employed to estimate the odds ratios (ORs) and 95% CIs of social-emotional problems of left-behind children with both migrated fathers and mothers compared with those with migrated fathers only after adjusting for county, children’s sex, age, and low birth weight, primary caregivers’ ethnicity, depression, assistant caregiving, and poor household wealth. We did not control for the primary caregivers’ age and education level because they were strongly collinear with the parental migration status (Table 1).

**Table 1:** Spearman’s correlation coefficients between variables of interest

	(1)	(2)	(3)	(4)	(5)	(6)	(7)	(8)	(9)
Parental migration status (1)	1.000								
County (2)	**−0.186**^a^	1.000							
Child sex (3)	0.024	−0.022	1.000						
Child age (4)	**0.299**^a^	−0.001	−0.020	1.000					
Low birthweight (5)	−0.009	0.015	0.012	0.045	1.000				
Caregiver ethnic (6)	−0.030	**0.210**^a^	0.040	−0.033	0.022	1.000			
Caregiver sex (7)	**0.222**^a^	**−0.137**^a^	0.036	**0.157**^a^	−0.011	−0.060	1.000		
Caregiver age (8)	**0.825**^a^	**−0.106**^a^	0.011	**0.316**^a^	0.006	−0.015	**0.277**^a^	1.000	
Caregiver education (9)	**−0.567**^a^	**0.087**^b^	0.053	**−0.156**^a^	−0.028	− 0.020	0.066	**− 0.473**^a^	1.000

^a^ Correlation is significant at the 0.01 level (2-tailed)

^b^ Correlation is significant at the 0.05 level (2-tailed)

Furthermore, stratified analysis was performed according to the groups of left-behind children. In each group of left-behind children, similar multivariable analyses were employed to examine the association of child age at the first parental migration, average duration per parental migration, and proportion of cumulative durations to parental migration in the child’s lifetime with early social-emotional problems, respectively, after controlling for county, children’s characteristics (sex, age, low birth weight), primary caregivers’ characteristics (sex, age, ethnic, education, and depression), assistant caregiving, and poor household wealth.

All analyses were performed using R software (Version 3.6.2). Two-sided *p* values < 0.05 were deemed to be statistically significant.

## Results

### Characteristics of the participants

Among the 811 left-behind children under 3 years, there were 287 (35.4%) FM-MC and 524 (64.6%) PM-GC. The characteristics of the left-behind children and their primary caregivers are summarized in Table 2. Among the left-behind children, the rate of low birth weight was 5.3%. Among their primary caregivers, the rate of depression reached 40.1%. Comparing the two groups (PM-GC vs FM-MC), there were significant differences for county, children’s age, and primary caregivers’ sex, age and education (*p* < 0.05).

**Table 2:** Characteristics of left-behind children and their primary caregivers

	Total	FM-MC	PM-GC	*p*-value
	(*n* = 811)	(*n* = 287 [35.4%])	(*n* = 524 [64.6%])	
**County, n (%)**				< 0.001
Yudu	230 (28.4)	44 (15.3)	186 (35.5)	
Sansui	271 (33.4)	107 (37.3)	164 (31.3)	
Tongjiang	181 (22.3)	80 (27.9)	101 (19.3)	
Lushi	78 (9.6)	26 (9.1)	52 (9.9)	
Pingshan	51 (6.3)	30 (10.5)	21 (4.0)	
**Children’s characteristics and measures**				
Boy, n (%)	436 (53.8)	159 (55.4)	277 (52.9)	0.488
Age (months), Median (IQR)	20 (13, 28)	16 (8, 24)	23 (16, 29)	< 0.001
Low birth weight, n (%)	43 (5.3)	16 (5.6)	27 (5.2)	0.797
**Primary caregiver’s characteristics and measures**				
Female, n (%)	744 (91.7)	287 (100.0)	457 (87.2)	< 0.001
Age (years), Median (IQR)	50 (30, 55)	28 (25, 31)	53 (50, 58)	< 0.001
Ethnic, n (%)				0.401
*Han*	635 (78.3)	220 (76.7)	415 (79.2)	
*Other*	176 (21.7)	67 (23.3)	109 (20.8)	
Education, n (%)				< 0.001
*Primary school or illiteracy*	404 (49.8)	33 (11.5)	371 (70.8)	
*Middle school*	296 (36.5)	167 (58.2)	129 (24.6)	
*High school or above*	111 (13.7)	87 (30.3)	24 (4.6)	
Having assistant caregiver(s), n (%)	566 (69.8)	200 (69.7)	366 (69.8)	0.962
Poor household wealth, n (%)	185 (22.8)	56 (19.5)	129 (24.6)	0.098
Depression, n (%)	325 (40.1)	107 (37.3)	218 (41.6)	0.230

FM-MC, left-behind children whose primary caregivers were their mothers with paternal (only father) migration; PM-GC, left-behind children whose primary caregivers were their grandparents with parental (both mother and father) migration

In this study, 759 left-behind children were evaluated for social-emotional development with the ASQ:SE. A total of 279 (36.8%) of the left-behind children had social-emotional problems (‘at risk’). The rate was 29.5% (78/264) in FM-MC and 40.6% (201/495) in PM-GC. Comparing the two groups (PM-GC vs FM-MC), the crude OR was 1.63 (95% CI: 1.18 to 2.24) and the adjusted OR was 1.51 (95% CI: 1.06 to 2.16) when adjusting for all covariates as shown in Table 2.

### Parent-child separation among left-behind children

Table 3 shows detailed descriptions of parent-child separation among left-behind children in different characteristics and groups. For paternal migration of all of these children, the medians of child’s age at the first migration and average duration per migration were 3 months (IQR: 1 to 9 months) and 4.48 months (IQR: 2.38 to 7.54 months), respectively; the proportion of cumulative migrating durations during the child’s lifetime was 0.72 (IQR: 0.49 to 0.88), which means that on average, left-behind children were separated from fathers for 72% of their life to date due to paternal migration. Compared with FM-MC, the child’s age at the first paternal migration and average duration per migration were higher in PM-GC (*p* < 0.05).

**Table 3:** The characteristics of parent-child separation in left-behind children

	Total	FM-MC	PM-GC	*p*-value
	(*n* = 811)	(*n* = 287)	(*n* = 524)	
Child age at the first paternal migration (months), Median (IQR)	3 (1, 9)	2 (1, 6)	5 (1, 12)	< 0.001
Child age at the first maternal migration (months), Median (IQR)	–	–	9 (6, 13)	–
Average duration per paternal migration (months), Median (IQR)	4.48 (2.38, 7.54)	3.39 (1.81, 5.57)	5.10 (3.07, 8.18)	< 0.001
Average duration per maternal migration (months), Median (IQR)	–	–	4.65 (2.71, 7.62)	–
Proportion of cumulative durations to paternal migration in the child’s lifetime, Median (IQR)	0.72 (0.49, 0.88)	0.73 (0.51, 0.87)	0.72 (0.47, 0.89)	0.670
Proportion of cumulative durations to maternal migration in the child’s lifetime, Median (IQR)	–	–	0.52 (0.28, 0.69)	–

FM-MC, left-behind children whose primary caregivers were their mothers with paternal (only father) migration; PM-GC, left-behind children whose primary caregivers were their grandparents with parental (both mother and father) migration

3 FM-MC and 21 PM-GC missed the information of child age (months) at the first paternal migration; 27 PM-GC missed the information of child age (months) at the first maternal migration; 12 FM-MC and 31 PM-GC missed the information of average duration per paternal migration and the proportion of cumulative durations to paternal migration in the child’s lifetime; 36 PM-GC missed the information of average duration per maternal migration and the proportion of cumulative durations to maternal migration in the child’s lifetime

For maternal migration of PM-GC, the medians of the child’s age at the first migration and average duration per migration were 9 months (IQR: 6 to 13 months) and 4.65 months (IQR: 2.71 to 7.62 months), respectively; the proportion of cumulative migrating durations in the child’s lifetime was 0.52 (IQR: 0.28 to 0.69), which means that PM-GC were separated from their mothers for 52% of their life to date due to maternal migration (Table 3).

**Fig. 1:**
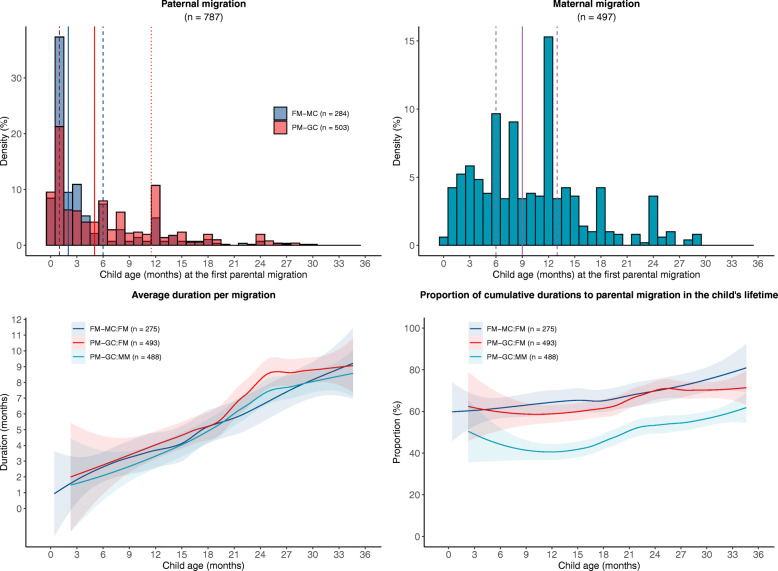
Description of paternal and maternal migration in different months of child age. FM, paternal migration; MM, parental migration; FM-MC, left-behind children whose primary caregivers were their mothers with paternal (only father) migration; PM-GC, left-behind children whose primary caregivers were their grandparents with parental (both mother and father) migration. Several left-behind children missed the information of characteristics of parental migration and the numbers of FM-MC and PM-GC with available data were marked in the figure

Paternal migration most often occurred when the child was 1 month old (27.1% of all left-behind children, 37.3% of FM-MC and 21.3% of PM-GC), and maternal migration most often occurred when the child was 12 months (15.3%), 6 months (9.7%) and 8 months (9.1%) (Fig. 1). Regardless of paternal migration of FM-MC (FM-MC: FM), paternal migration of PM-GC (PM-GC: FM), or maternal migration of PM-GC (PM-GC: MM), with increasing child age, growing trends can be observed in the average duration per migration and the proportion of cumulative durations in the child’s lifetime (Fig. 1).

### Association between parent-child separation and the children’s social-emotional problems

Table 4 shows the association of different characteristics of parent-child separation with the children’s social-emotional problems in different groups. In both FM-MC and PM-GC, no significant association was observed between any feature of paternal migration and the children’s social-emotional problems. However, in PM-GC, the children’s social-emotional problems were significantly associated with the average duration per maternal migration (OR: 1.05; 95% CI: 1.01 to 1.09) and the proportion of cumulative durations of maternal migration in the child’s lifetime (OR: 2.98; 95% CI: 1.36 to 6.51). After adjustment for county, children’s characteristics (sex, age, low birth weight), primary caregivers’ characteristics (age, ethnicity, education, depression, having assistant caregiver) and poor household wealth, the children’s social-emotional problems were only significantly associated with the proportion of cumulative durations of maternal migration in the child’s lifetime (aOR: 2.83; 95% CI: 1.13 to 7.10).

**Table 4:** Association between parent-child separation characteristics and child’s social-emotional problems

	OR (95% CI)	aOR (95% CI)
**FM-MC**		
Child age at the first paternal migration	0.96 (0.91 to 1.02)	0.96 (0.90 to 1.02)
Average duration per paternal migration	1.03 (0.99 to 1.09)	1.00 (0.94 to 1.05)
Proportion of cumulative durations to paternal migration in the child’s lifetime	1.29 (0.44 to 3.82)	0.90 (0.26 to 3.08)
**PM-GC**		
Child age at the first paternal migration	1.01 (0.98 to 1.04)	1.00 (0.97 to 1.03)
Child age at the first maternal migration	0.98 (0.95 to 1.01)	0.97 (0.94 to 1.01)
Average duration per paternal migration	1.00 (0.97 to 1.03)	0.96 (0.93 to 1.00)
Average duration per maternal migration	**1.05 (1.01 to 1.09)**	1.01 (0.96 to 1.05)
Proportion of cumulative durations to paternal migration in the child’s lifetime	0.99 (0.49 to 2.02)	0.98 (0.42 to 2.29)
Proportion of cumulative durations to maternal migration in the child’s lifetime	**2.98 (1.36 to 6.51)**	**2.83 (1.13 to 7.10)**

ORs were not adjusted for any covariate; aORs were adjusted for county, children’s characteristics (sex, age, low birth weight), primary caregivers’ characteristics (age, ethnic, education, depression, having assistant caregiver), and poor household wealth. The bolding ORs or aORs are statistically significant at the level of *p* < 0.05

## Discussion

Parent-child separation among left-behind children is regarded as one of the largest adversities in early childhood and may have both short-term and long-term effects on learning, behaviour, and both physical and mental well-being [2, 25–27]. In addition to a decreasing-age trend of parent-child separation, which has been reported by previous research [1, 28], this study described more detailed characteristics of parent-child separation among under-3 left-behind children in poor rural China.

We found that paternal migration occurred earlier and longer than maternal migration. Half of the left-behind children separated from their fathers before 3 months of age, whereas half separated from their mothers before 9 months of age. The later maternal migration may be attributed to breastfeeding in the early months and a traditional postpartum practice called ‘sitting the month’ (‘Zuò yuè zi’ in Chinese), which means mothers are expected to rest indoors for 1 month or 40 days after giving birth [29, 30].

On average, during the first 3 years of life, left-behind children were separated from fathers for 72% of their life and from mothers for 52% of their lifetime at the time of the survey. The average and cumulative duration of both paternal and maternal migration gradually increased with increasing child age. During the first 3 years, the most critical period of growth and development in early life, such early-initiating and long-lasting parental migration is a big threat to many aspects of left-behind children’s health and well-being, despite the increase of household incomes that is often linked with positive health outcomes of children [2, 31].

Many previous studies have indicated that left-behind children have a higher risk of social-emotional problems than children who were not left behind [32–34]. In this study, the prevalence of social-emotional problems among under-3 left-behind children in poor rural areas was 36.8%, which was higher than the 23.0% reported in a previous cross-sectional survey conducted in poor rural areas of Shanxi and Guizhou by the UNICEF in 2013 [33]. It is worth paying more attention to the upward trend of social-emotional problems among left-behind children in poor rural areas. Moreover, we also found a quite high prevalence (40.1%) of depression among their primary caregivers. Several studies have highlighted the psychological problems of caregivers in rural China. In repeated cross-sectional surveys conducted by UNICEF across six counties of northern and southern China, the prevalence of depression in caregivers of rural children aged 6–35 months with non-migrant parents, migrant fathers, and both migrant parents was found to be 40.0, 37.1, and 50.6% in 2013 and 35.9, 31.8, and 38.0% in 2016, respectively [4]. Previous studies have reported an association between caregivers’ depressive symptoms and social-emotional problems, probably mediated by decreased responsive parenting, lower levels of daily stimulation and acceptance, and other aspects found in poor home environments [22, 31, 35, 36]. Therefore, when implementing interventions for social-emotional development among left-behind children, it is necessary to improve the caregivers’ mental health and skills to nurture a friendly home environment for left-behind children.

This study also found that children with both fathers and mothers who migrated were more likely to have social-emotional problems than those with migrated fathers only (40.6% vs 29.5%). Our analysis of multiple features of parental migration further shows that paternal migration had no association with the children’s social-emotional development. This was consistent with no association between paternal migration and early child development reported by previous research in China and other countries [37–39]. For maternal migration, the left-behind children’s social-emotional problems were significantly linked to the proportion of cumulative migrating duration in the child’s lifetime, but with no significance for the child’s age in months at the first migration or the average migrating duration. This indicates that cumulative exposure to maternal migration may be negatively associated with children’s social-emotional development.

Our previous research found that the high risk of social-emotional problems among left-behind children in early childhood may be primarily caused by the transition of family structure and function and the consequently weakened home environment, such as increased violent discipline, decreased age-appropriate stimulation and responsive care [9]. Interrupted infant-mother attachment may also play a role in the pathway linking maternal migration and a higher risk of social-emotional problems. The attachment pattern of children with their parents, especially their mothers, begins to develop as early as in the first 2 months of life and is regarded as a central aspect of social and emotional development [40]. Parental migration in early childhood interrupts the development of parent-child attachment. Left-behind children have to experience anxiety due to such separation and having to form a new relationship with other caregivers. However, the establishment of attachment with other caregivers cannot completely replace the absence of parent-child attachment, especially maternal attachment [41]. The disruption of infant-mother attachment could weaken their affection, causing a high level of insecurity [42, 43], and disturb the development of language, behaviours, and social-emotional functions [41, 44–48].

It should be emphasized that despite differences across children with various parental migrations, social-emotional delays are a widespread problem in rural China and affect not only left behind children but also all children [33, 49, 50]. Additionally, a high prevalence of SE delays was also found to exist across wide regions of rural China and has been reported by different research groups when using different scales for the measurement of SE skills [28, 32–34, 49–54]. A multidimensional intervention framework is needed to support all rural children as well as their families, especially those with both migrant parents.

In our previous study, we found that communication between migrated parents and caregivers is associated with reduced caregiver depression and improved nurturing care, thus helping to reduce the risk of social-emotional problems in left-behind children [9]. Therefore, when parental migration is inevitable, early childhood development programs are recommended to encourage parent-caregiver communication, support the caregivers’ mental health, and guide nurture care practices for achieving full developmental potential.

### Limitations of the study

This study has several limitations. First, there may be information bias when recalling the time of parent-child migration, so we asked the primary caregivers to report the child’s age of months at each time of paternal or/and maternal migration to help them recall more conveniently. Second, the risk of social-emotional problems may be overestimated because the ASQ:SE used in this study is a screening tool. Third, this study only included the two most common types of left-behind children: one type/group was those with only paternal migration and maternal caregiving; the other type/group was those with both paternal and maternal migrations and grandparental caregiving. Other types were excluded due to their small sample size, such as children who were left behind by only their fathers but not cared for by their mothers. Further research on vulnerable left-behind children is needed to explore their characteristics, determinants and effective interventions to improve their social-emotional development.

## Conclusion

This study shows a high risk of social-emotional problems among left-behind children under 3 years of age in poor rural China and highlights that cumulative exposure to maternal migration may be detrimental to left-behind children’s early social-emotional development. Maternal care is strongly recommended for children in early childhood, and programs for early child development are needed to support left-behind children as well as their families. Further research is warranted to explore the mechanism of parental migration affecting the well-being of left-behind children and to develop effective intervention strategies.

## Supplementary Information

**Additional file 1.** Questionnaire for Basic Information and Parental Migration Status of Left-behind Children.

## Data Availability

The datasets generated and/or analyzed during the current study are not publicly available due to the restrictions of the local ethnics committee and institutional data security and privacy policies. The data are accessible from the corresponding author (Prof Jingxu Zhang, jxzhang@bjmu.edu.cn) on reasonable request and after obtaining institutional and ethics committee’s approval.

## Abbreviations

aOR: Adjusted odds ratio
ASQ:SE: Ages and stages questionnaire: social-emotional (ASQ:SE)
FM-MC: Children who were left behind by fathers and cared for by mothers
IQRs: Inter-quartile range
LBC: Left-behind children
LMICs: Low-income or middle-income countries
PM-GC: Children who were left behind by both parents and cared for by grandparents
RLBCHD: Rural left-behind children health and development programme
UNICEF: United Nations children’s fund
ZSDS: Zung’s self-rating depression scale
