# Supplementary material for: Impact of parent-child separation on children’s social-emotional development: a cross-sectional study of left-behind children in poor rural areas of China
Source: BMC Public Health. 2021 Apr 29;21:823. doi: 10.1186/s12889-021-10831-8 (PMC8082618; doi:10.1186/s12889-021-10831-8)
Supplement: Supplementary file 1 — Additional file 1. Questionnaire for Basic Information and Parental Migration Status of Left-behind Children. [file 12889_2021_10831_MOESM1_ESM.docx]

| **Questionnaire for Basic Information and Parental Migration Status of Left-behind Children** | | | |
| --- | --- | --- | --- |
| **FDI1** | **County** | | |
|  | 1. Lushi 2. Pingshan 3. Yudu 4. Sansui 5. Tongjiang | | |
| **FDI2** | **Township/Street：________________________** | | |
| **FDI3** | **Village：________________________** | | |
| **INo** | **Name of investigator：________________________** | | |
| **ITIME** | **Date of investigation：□□□□Year□□Month□□Day** | | |
| **Sociodemographic Characteristics of the Child (CSD)** | | | |
| **CSD2** | **Sex of the child** | | |
|  | 1. Boy 2. Girl | | |
| **CSD3** | **Birth date of the child (solar calendar)：□□□□****Year□□Month□□Day** | | |
| **Child birth(BR)** | | | |
| **BR1** | **Delivery mode of the child** | | |
|  | 1. Vaginal delivery 2. Cesarean section 3.Unknown | | |
| **BR2** | **Gestational age at birth** | | |
|  | 1. Preterm (gestational age of < 37 weeks) 2. full-term (gestational age of 37-41 weeks) (*Skipping to BR4*) 3. post term (*Skipping to BR4*) 4.Unknown | | |
| **BR3** | **If the child was born prematurely, how many days before the expected date of childbirth? □□□□Day** | | |
| **BR4** | **Birthweight: □□□□.□□g** (*If you don't know the birthweight, fill in – 9*) | | |
| **BR5** | **Was the child hospitalized within one month after birth due to suffering the following diseases?** | | |
|  | 1. No 2. Perinatal asphyxia 3. Intracranial hemorrhage 4. Pathological jaundice 5. Meningitis 6. Convulsion 7. Others, specifically_______ | | |
| **Family Structure and Members(FSM)** | | | |
| **FSM4** | **Family members who are now living with the child** (*including migrated parents who are not at home*) | | |
|  | 1. Mother 2. Father 3. Grandfather 4. Grandmother 5. Maternal grandfather 6. Maternal grandmother 7. Uncle 8. Aunt 9. Older brother ___ persons **FSM4_9** 10. Younger brother ___ persons[**FSM_10**] 11. Elder sister ___ persons**[FSM_11]** 12. Younger sister ___ persons**[FSM4_12]** 13. Sister/Brother in law 14. Sister/Brother in law 15. Other family members ___ persons | | |
| **FSM5** | **The reasons why the mother don't live with the child** (*If FSM4 did not select mother, load and ask this question*) | | |
|  | 1. Divorce/separation from the father/single parent 2. Death 3. Other reasons | | |
| **FSM5a** | **How old was the child when he/she no longer lives with his/her mother? □□Months** (*If FSM4 did not select mother, load and ask this question*) | | |
| **FSM6** | **The reasons why the father don't live with the child** (*If FSM4 did not select father*) | | |
|  | 1. Divorce/separation from the father/single parent 2. Death 3. Other reasons | | |
| **FSM6a** | **How old was the child when he/she no longer lives with his/her father? □□Months** (*If FSM4 did not select father FDI4-2*) | | |
| **Caregiving Arrangement (CR)** | | | |
| **CR1** | **Are you the child’s** | | |
|  | 1. Mother(*Skipping to PSD3*) 2. Father 3. Grandfather 4. Grandmother 5. Maternal grandfather 6. Maternal grandmother 7. Other | | |
| **CR2** | **Are you the child’s primary caregiver?** | | |
|  | 1. Yes (*Skipping to CR4*) 2. No | | |
| **CR3** | **If not, Who is the child’s primary caregiver?** (*If CR2 choose No, ask this question, and try to contact with the primary caregiver*) | | |
|  | 1. Mother 2. Father 3. Grandfather 4. Grandmother 5. Maternal grandfather 6. Maternal grandmother 7. Other family members | | |
| **CR4** | **Is there anyone else to take care of your child?** | | |
|  | 1. No 2. Grandfather 3. Grandmother 4. Maternal grandfather 5. Maternal grandmother 6. Mother 7. Father 8. Other family members | | |
| **Sociodemographic Characteristics of the Primary Caregiver (PSD)** | | | |
| **PSD3** | **Solar birth date of the primary caregiver: □□□□Year□□Month□□Day** | | |
| **PSD4** | **Sex of the primary caregiver** | | |
|  | 1. Male 2. Female | | |
| **PSD5** | **Ethnic origin of the primary caregiver** | | |
|  | 1. Han 2. Hui 3. Man 4. Menggu 5. Zang 6. Miao 7. Dong 8. Zhuang 9. Tujia 10. Yao 11. Other: ____ | | |
| **PSD6** | **Education of the primary caregiver** | | |
|  | 1. Illiteracy 2. Primary school 1-3 years 3. Primary school 4-6 years 5. Middle school 6. High school 7. Technical secondary school 8. Junior college 9. Bachelor degree or above | | |
| **Maternal Migration Status (MM)** | | | |
|  | *If FSM4-1 ≠ 1, that is, when the mother is no longer living with the child, the module of* ***Maternal Migration Status*** *will no longer be loaded.* | | |
| **MM1** | **The reason why the child's mother do not come here or is not home** **(If CR1≠1&CR2=1, ask this question)** | | |
|  | 1.Migration for work 2. Divorce/separation from the father/single parent 3. Illness or pregnancy 4. Other reasons | | |
| **MM2** | **How long the last migration of the mother? □□Months□□Days(If MM1=1, ask this question)** | | |
|  | 1. < 7 days 2. 7 days ~ 29 days 3. 30 days ~ 89 days 4. 90 days ~ 179 days 5. 180 days ~ 364 days 6. ≥ 365 days | | |
| **MM3** | **Had the mother leave the child home and migrated for work for more than one week before this migration?** | | |
|  | 1. Yes 2. No (*Skipping to MM7*) 3.Unknown (*Skipping to MM7*) | | |
| **MM4** | **How old was the child at the first migration of his/her mother? □□Months** | | |
| **MM5** | **How many times did the migrated mother come home after the first migration****? □□Times** | | |
| **MM6** | **On average, how long did the mother stay at home when she returns home after migration?** **□□****□□Days** | | |
| **MM7** | **Does the mother contact you (the primary caregiver) when she migrated out for work?** | | |
|  | 1. Yes 2. No (*Skipping to FM1*) 3. Unsuitable (*Skipping to FM1*) | | |
| **MM8** | **What is the main method of contact between the migrated mother and the primary caregiver?** | | |
|  | 1. Phone call 2. Video call 3. Other | | |
| **MM9** | **How often does the migrated mother have contact with you (the primary caregiver)?** | | |
|  | 1. 1 time/day 2. 4~6 times/week 3. 1~3 times/week 4. 2~3 times/month 5. 1 time/month 6. < 1 time/month | | |
| **MM10** | **How long is the average duration per communication between the migrated mother and you (the primary caregiver)?** | | |
| **MN11** | **How often does the migrated mother communicate with the child?** | | |
|  | 1. Frequently 2. Sometimes 3. Never | | |
| **MM12** | **How often does the migrated mother talk with you about how to take care of the child during each contact?** | | |
|  | 1. Frequently 2. Sometimes 3. Never | | |
| **MM13** | **Does the migrated mother ask about your (the primary caregiver) health condition at each contact?** | | |
|  | 1. Yes 2. No | | |
| **Paternal Migration Status (FM)** | | | |
|  | *If FSM4-2 ≠ 1, that is, when the father is no longer living with the child, the module of* ***Paternal Migration Status*** *will no longer be loaded.* | | |
| **FM1** | **Is the father at home?** (*If CR1≠2&CR2=1, ask this question*) | | |
|  | 1.Migration for work 2. Divorce/separation from the mother/single parent 3. Illness or pregnancy 4. Being at home | | |
| **FM2** | **How long the last migration of the father? □□□□Days** (*If FM1=1, ask this question*) | | |
|  | 1. < 7 days 2. 7 days ~ 29 days 3. 30 days ~ 89 days 4. 90 days ~ 179 days 5. 180 days ~ 364 days 6. ≥ 365 days | | |
| **FM3** | **Had the father leave the child home and migrated for work for more than one week before this migration?** | | |
|  | 1. Yes 2. No (*Skipping to FM7*) 3.Unknown (*Skipping to FM7*) | | |
| **FM4** | **How old was the child at the first migration of his/her father? □□Months** | | |
| **FM5** | **How many times did the migrated father come home after the first migration? □□Times** | | |
| **FM6** | **On average, how long did the father stay at home when she returns home after migration? □□Months□□Days** | | |
| **FM7** | **Does the father contact you (the primary caregiver) when she migrated out for work?** | | |
|  | 1. Yes 2. No(Skipping to FE1) 3. Unsuitable (Skipping to FE1) | | |
| **FM8** | **What is the main method of contact between the migrated father and the primary caregiver** | | |
|  | 1. Phone call 2. Video call 3. Other | | |
| **FM9** | **How often does the migrated father have contact with you (the primary caregiver)?** | | |
|  | 1. 1 time/day 2. 4~6 times/week 3. 1~3 times/week 4. 2~3 times/month 5. 1 time/month 6. < 1 time/month | | |
| **FM10** | **How long is the average duration per communication between the migrated father and you (the primary caregiver)?** | | |
| **FM11** | **How often does the migrated father communicate with the child?** | | |
|  | 1. Frequently 2. Sometimes 3. Never | | |
| **FM12** | **How often does the migrated father talk with you about how to take care of the child during each communication?** | | |
|  | 1. Frequently 2. Sometimes 3. Never | | |
| **FM13** | **Does the migrated father ask about your (the primary caregiver) health condition at each contact?** | | |
|  | 1. Yes 2. No | | |
| **Family Economics (FE)** | | | |
| **FE2** | **Do you (the primary caregiver) have a smart phone (a phone can make you use Wechat and QQ, etc.)?** | | |
|  | 1. Yes 2. No | | |
| **FE3** | **Can you (the primary caregiver) surf the internet at home?** | | |
|  | 1. Yes 2. No | | |
| **FE4** | **Household electrical appliances and vehicles owned?** | | |
|  | 1. Automobile |  |  |
|  | 2. Motorcycle |  |  |
|  | 3. Tractor/ Tricycle |  |  |
|  | 4. TV |  |  |
|  | 5. Air conditioner |  |  |
|  | 6. Washing machine |  |  |
|  | 7. Refrigerator |  |  |
|  | 8. PAD/ Computer |  |  |
|  | 9. Rice cooker |  |  |
